# Unplanned readmission prevention by a geriatric emergency network for transitional care (URGENT): a prospective before-after study

**DOI:** 10.1186/s12877-019-1233-9

**Published:** 2019-08-07

**Authors:** Pieter Heeren, Els Devriendt, Steffen Fieuws, Nathalie I. H. Wellens, Mieke Deschodt, Johan Flamaing, Marc Sabbe, Koen Milisen

**Affiliations:** 1 Department of Public Health and Primary Care, Academic Centre for Nursing and Midwifery, KU Leuven, Kapucijnenvoer 35/4, 3000 Leuven, Belgium; 2 Department of Geriatric Medicine, University Hospitals Leuven, Herestraat 49, 3000 Leuven, Belgium; 3 Research Foundation Flanders, Egmontstraat 5, 1000 Brussels, Belgium; 4 I-Biostat, Interuniversity Institute for Biostatistics and statistical Bioinformatics KU Leuven, Kapucijnenvoer 35/3, 3000 Leuven, Belgium; 5 Public Health and Social Affairs Department, Government Canton Vaud, Avenue des Casernes 2, 1014 Lausanne, Switzerland; 6 Department of Chronic Diseases, Metabolism and Ageing, Gerontology and Geriatrics, KU Leuven, Herestraat 49, 3000 Leuven, Belgium; 7 Department of Public Health, Nursing Science, University of Basel, Bernoullistrasse 28, 4056 Basel, Switzerland; 8 Department of Emergency Medicine, University Hospitals Leuven, Herestraat 49, 3000 Leuven, Belgium; 9 Department of Public Health and Primary Care, Emergency Medicine, KU Leuven, Kapucijnenvoer 35/4, 3000 Leuven, Belgium

**Keywords:** Emergency department, Geriatric care model, Comprehensive geriatric assessment, Unplanned ED readmission, Nurse-led

## Abstract

**Background:**

URGENT is a comprehensive geriatric assessment (CGA) based nurse-led care model in the emergency department (ED) with geriatric follow-up after ED discharge aiming to prevent unplanned ED readmissions.

**Methods:**

A quasi-experimental study (sequential design with two cohorts) was conducted in the ED of University Hospitals Leuven (Belgium). Dutch-speaking, community-dwelling ED patients aged 70 years or older were eligible for enrolment. Patients in the control cohort received usual care. Patient in the intervention cohort received the URGENT care model.

A geriatric emergency nurse conducted CGA and interdisciplinary care planning among older patients identified as at risk for adverse events (e.g. unplanned ED readmission, functional decline) with the interRAI ED Screener© and clinical judgement of ED staff. Case manager follow-up was offered to at risk patients without hospitalization after index ED visit. For inpatients, geriatric follow-up was guaranteed on an acute geriatric ward or by the inpatient geriatric consultation team on a non-geriatric ward if considered necessary.

Primary outcome was unplanned 90-day ED readmission. Secondary outcomes were ED length of stay (LOS), hospitalization rate, in-hospital LOS, 90-day higher level of care, 90-day functional decline and 90-day post-hospitalization mortality.

**Results:**

Almost half of intervention patients (404/886 = 45.6%) were categorized at risk. These received on average seven advices. Adherence rate to advices on the ED, during hospitalization and in community care was 86.1, 74.6 and 34.1%, respectively. One out of four at risk patients without hospitalization after index ED visit accepted case manager follow-up. Unplanned ED readmission occurred in 170 of 768 (22.1%) control patients and in 205 of 857 (23.9%) intervention patients (*p* = .11). The intervention group had shorter ED LOS (12.7 h versus 19.1 h in the control group; *p* < .001), but higher rate of hospitalization (70.0% versus 67.0% in the control group; *p* = .003).

**Conclusions:**

The URGENT care model shortened ED LOS and increased the hospitalization rate, but did not prevent unplanned ED readmissions. A geriatric emergency nurse could improve in-hospital patient management, but failed to introduce substantial out-hospital case-management.

**Trial registration:**

The protocol of this study was registered retrospectively with ISRCTN (ISRCTN91449949; registered 20 June 2017).

**Electronic supplementary material:**

The online version of this article (10.1186/s12877-019-1233-9) contains supplementary material, which is available to authorized users.

## Background

The growing group of older adults has become an important subset of emergency department (ED) patients with already 12–24% of ED admissions being persons aged 65 years or over [1]. This evolution results in increasing patient volumes in a system that is already burdened with crowding [2] and yields a qualitative challenge, as well, because older adults are characterized by vulnerability features, such as decreased physiological reserves, presence of geriatric syndromes (e.g. delirium), multimorbidity with polypharmacy and potential atypical disease presentation. In addition, other factors such as pre-existing functional impairment, cognitive decline and social issues hamper disposition planning. It is obvious that managing these patients in a fast-paced environment is challenging [2, 3]. This is reflected in poorer outcomes regarding functional decline, hospitalization and return rates and death in older adults, compared with younger patients [4]. For example, up to one out of four older adults return to the ED within 3 months [4, 5]

To improve the outcomes of older ED patients, international guidelines recommend adapting the classic disease-oriented ED approach towards a comprehensive patient-oriented approach [6, 7]. Implementing comprehensive geriatric assessment (CGA) in the ED is promoted in that respect. CGA has been defined “a multidimensional interdisciplinary diagnostic process focused on determining a frail older person’s medical, psychosocial and functional capabilities in order to develop a coordinated and integrated plan for treatment and long-term follow-up” [8]. Although this approach has been proven effective in acute geriatric wards, [9] its effectiveness in ED-based care models remains inconclusive due to inconsistent study results and several methodological issues, such as non-transparent reporting of intervention processes (e.g. fidelity to CGA-based advices) [10].

To stimulate continuity of care, transitional care models are promoted [6, 7]. These combine ED-based CGA with structured follow-up after ED discharge. ‘Unplanned Readmission prevention by Geriatric Emergency Network for Transitional care’ (URGENT) is an example of such a transitional care model that was developed and implemented in the ED and region of University Hospitals Leuven in Belgium [11]. This manuscript reports the evaluation of the URGENT care model.

## Methods

For detailed information concerning the study setting, care model development and methods, the authors refer to the study protocol that was registered retrospectively with ISRCTN (ISCRCTN91449949) [11]. As mentioned in the protocol paper, the aim of this study is to evaluate the effectiveness of the URGENT care model compared to usual care on the unplanned ED readmission rate, as primary outcome, and secondary outcomes (i.e. ED length of stay (LOS), hospitalization rate, in-hospital LOS, higher level of care, functional decline and post-hospitalization mortality) of community-dwelling, older adults.

### Design

A single-center, quasi-experimental before-after study was conducted. This yielded a sequential design with two cohorts, comparing usual ED care in the control cohort (CC) to the URGENT care model in the intervention cohort (IC). Patients were recruited from December 1, 2014 to May 31, 2015 (CC) and from October 15, 2015 to May 31, 2016 (IC). Between these two cohorts, a 4-month period was used to pilot and implement the URGENT care model. Patient data collection was completed on August 31, 2016.

### Participants

This study targeted Dutch-speaking, community-dwelling ED patients aged 70 years or older. Exclusion criteria were living in a residential care setting, being transferred to the ED from an inpatient ward or another hospital, having a medical condition that makes an interview impossible, being unable to give informed (proxy) consent or being admitted to the ED on Saturday.

### Intervention

The URGENT care model was led by a dedicated geriatric emergency nurse that added four consecutive steps to usual ED care. First, older patients at risk for adverse events (i.e. unplanned ED readmission, long ED LOS, hospitalization, long in-hospital LOS, higher level of care, functional decline and post-hospitalization mortality) were identified with two methods; the interRAI ED screener© [12] (iEDS) and clinical judgement. Patients stratified with a high risk (iEDS score 5–6) were offered the subsequent steps of the intervention. Patients stratified with a low risk (iEDS score 1–4) were offered the subsequent steps if someone of the ED staff estimated that the patient might benefit from it (i.e. clinical judgement). Second, the geriatric emergency nurse conducted CGA among patients identified at risk for adverse events. Third, a CGA-based interdisciplinary care plan was tailored to the patient’s needs, capacity and preferences. Fourth, geriatric follow-up was provided if necessary. For inpatients, geriatric follow-up was guaranteed on an acute geriatric ward or by the inpatient geriatric consultation team on a non-geriatric ward if considered necessary [13]. For patients identified as at risk and without hospitalization after index ED visit; case manager follow-up by a community nurse or social worker was planned. Case management comprised free-of-charge assistance and support with the implementation of CGA-based advices after ED discharge. In addition, the geriatric emergency nurse was authorized to refer patients autonomously to the geriatric day clinic of University Hospitals Leuven for specialized in-depth assessment (e.g. falls, cognitive problems) and medical evaluation.

### Outcome measures

The primary outcome was 90-day unplanned ED readmission. Secondary outcomes were hospitalization rate, ED LOS, in-hospital LOS, higher level of care (i.e. a professionally organized living arrangement that differs from the patient’s usual living place in the community), functional decline (i.e. an increase of 2 or more points on the total 6-item Katz activities of daily living (ADL) score [14]) and post-hospitalization mortality. All post-ED outcomes were measured 30 and 90 days after hospital discharge. Higher level of care was also measured at hospital discharge. Functional decline and higher level of care at 30 and 90 days after hospital discharge were only measured among patients without hospitalization after index ED visit.

### Covariates

Following patient characteristics were registered at baseline with semi-structured interview and electronic patient file review: gender, age, living situation, triage priority level (Emergency Severity Index [15] (ESI)), first treating discipline on the ED, ED specific geriatric screening (iEDS [12]), ADL (6-item KATZ-scale [14]), fall history [12], pain perception [12], weight loss [12], caregiver burden [12], dependence in instrumental activities of daily living (IADL), polypharmacy, cognition (Mini-Cog [16], Confusion Assessment Method [17] (CAM)), screening for depression [18], comorbidity score (Cumulative Illness Rating Scale [19] (CIRS)) and previous ED visit and hospital stay during the 90 days before index ED visit .

### Procedure

Study nurses recruited patients and collected baseline data of all CC patients and of IC patients at low risk for adverse events. In the IC, a geriatric emergency nurse scored the iEDS, discussed clinical judgement with ED staff (e.g. nurse, physician or social worker) and collected baseline data of patients at risk for adverse events. In both cohorts, study nurses performed outcome registration by review of the electronic patient file and by telephone calls. The latter were only performed among patients without hospitalization after index ED visit.

### Statistical analyses

Required sample size based on a two-sided test (with α =0.05 and at least 80% power) was 751 patients per cohort, making a total of 1502 patients. This calculation was based on the assumption of a 12-weeks unplanned ED readmission rate of 27% among hospitalized patients and 23% among non-hospitalized patients, a hospitalization rate of 70% and a 25% relative reduction of readmission rates. All analyses have been performed using SAS software, version 9.4 of the SAS System for Windows. *P*-values smaller than 0.05 were considered significant. Cause-specific hazard-ratios, relative risk ratios, odds ratios and ratios of geometric means comparing IC with CC were reported when appropriate. In all comparative analyses of outcome measures, propensity scores (applying inverse probability of treatment weighting) were used to handle the potential differences in patient mix between the cohorts [20, 21]. Bonferroni-Holm correction was applied if relevant. For more details, we refer to our protocol paper [11].

## Results

### Study samples and patient characteristics

**Fig. 1:**
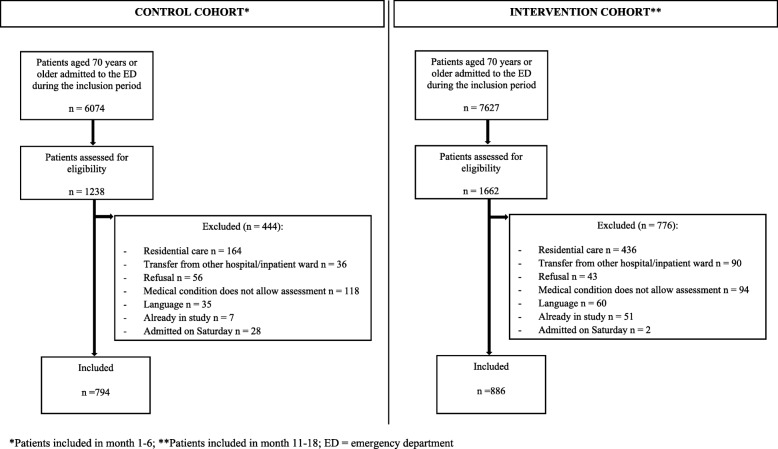
Overview of the study sample

During the recruitment periods, 2900 individuals were screened for eligibility. Of these individuals, 1220 were excluded, resulting in a sample of 794 CC patients (Additional file 1) and a sample of 886 IC patients (Fig. 1). There were no deaths during the index ED visit. During hospitalization following index ED stay, 26 CC patients and 29 IC patients died. Inpatient deaths were excluded from outcome analyses, except for following: hospitalization rate, ED LOS and in-hospital LOS.

Table 1 describes the characteristics of the CC and IC patients. CC patients had more comorbidities (*p* < 0.001), got more often a high risk geriatric screening score (*p* < 0.001) and reported more frequent caregiver burden (*p* = 0.02), unintended weight loss in the preadmission period (p < 0.001) and increased depression risk (*p* = 0.001). IC patients had more often difficulties in medication management (*p* = 0.01), reported more frequent help for finances (p = 0.02) and visited the ED more often in the 90-day preadmission period (*p* = 0.03).

**Table 1:** Patient characteristics

Variable	All patients (*n* = 1680)	Control cohort (*n* = 794)	Intervention cohort (*n* = 886)	*P*-value
Age, Q2 (Q1-Q3)	80 (76.0–85.0)	80 (75.0–85.0)	81 (76.0–85.0)	0.46
Female, n (%)	905 (53.9)	436 (54.9)	469 (52.9)	0.42
Living alone, n (%)	610 (36.4)	303 (38.2)	307 (34.8)	0.15
Nursing care at home, n (%)	552 (33.0)	250 (31.6)	302 (34.3)	0.25
Home care, n (%)	246 (14.7)	120 (15.1)	126 (14.3)	0.64
Physiotherapy, n (%)	199 (11.9)	103 (13.0)	96 (10.9)	0.19
Meals on wheels, n (%)	195 (11.7)	92 (11.6)	103 (11.7)	0.95
Cleaning help, n (%)	745 (44.6)	355 (44.8)	390 (44.4)	0.87
Help for management of finances, n (%)	565 (33.8)	246 (31.0)	319 (36.3)	0.02*
Personal alarm system, n (%)	183 (11.0)	79 (10.0)	104 (11.8)	0.22
Caregiver burden, n (%)	206 (12.5)	114 (14.4)	92 (10.7)	0.02*
Premorbid ADL, Q2 (Q1-Q3)	7 (6.0–10.0)	7 (6.0–10.0)	7 (6.0–10.0)	0.06
Fall in last 90 days, n (%)	679 (40.7)	341 (43.2)	338 (38.5)	0.06
Pain, daily and severe, n (%)	247 (14.8)	110 (13.9)	137 (15.6)	0.33
Weight loss, n (%)	309 (18.6)	180 (22.8)	129 (14.8)	<.001*
Difficulty in medication management, n (%)	353 (21.2)	146 (18.4)	207 (23.6)	0.01*
Cognitive impairment, n (%)	706 (46.7)	355 (46.8)	351 (46.6)	0.93
Delirium, n (%)	66 (3.9)	28 (3.5)	38 (4.3)	0.42
Risk for depression, n (%)	273 (16.4)	175 (22.2)	98 (11.2)	.001*
Polypharmacy, n (%)	1201 (72.4)	565 (71.2)	636 (73.6)	0.26
Triage priority level (ESI), n (%)				0.26
°level 1–2	547 (37.2)	250 (35.7)	297 (38.6)	
°level 3–5	923 (62.8)	450 (64.3)	473 (61.4)	
Comorbidity, Q2 (Q1-Q3)	19 (15.0–24.0)	21 (16.0–26.0)	18 (14.0–23.0)	<.001*
Previous ED visit in last 90 days, n (%)	381 (23.0)	162 (20.6)	219 (25.1)	0.03*
Previous hospital stay in last 90 days, n (%)	394 (23.6)	180 (22.8)	214 (24.3)	0.49
First treating discipline on ED is surgical, n (%)	351 (20.9)	175 (22.0)	176 (19.9)	0.27
Geriatric screening (iEDS), n (%)				<.001*
°Score 1–4	1163 (69.3)	506 (63.9)	657 (74.2)	
°Score 5–6	515 (30.7)	286 (36.1)	229 (25.8)	

*P*-value: comparison of variable between control and intervention cohort, using Mann-Whitney U or Chi^2^ tests

*statistical significant with alpha = 0,05, *ED* emergency department, *ESI* Emergency Severity Index, *ADL* Activities of Daily Living; Q2 = median; Q1-Q3 = interquartile range; iEDS = interRAI Emergency Department Screener©

### Patients labelled at risk in the intervention cohort

**Fig. 2:**
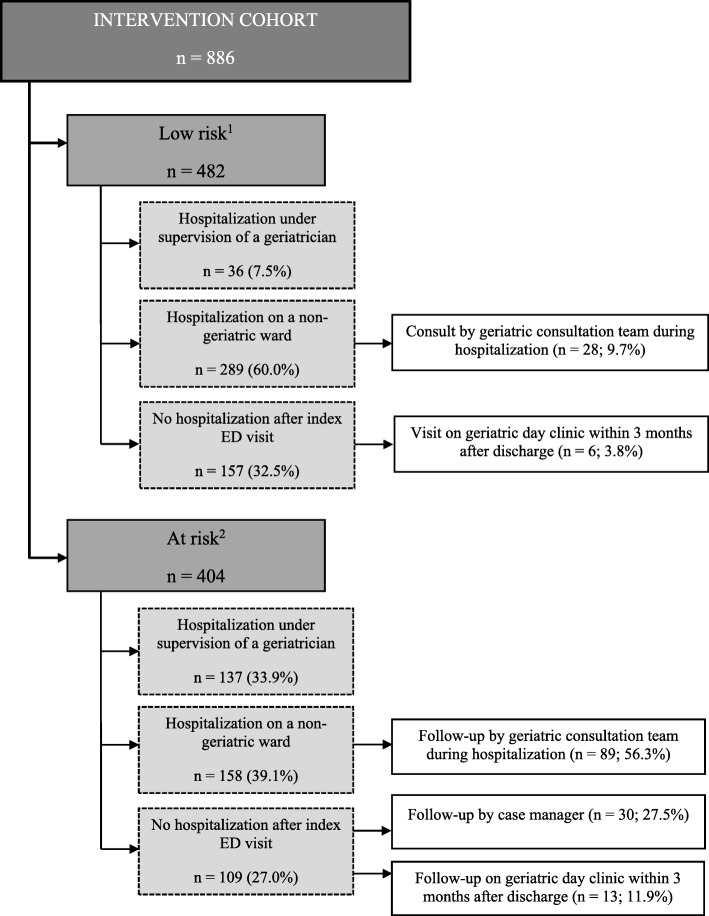
Overview of the intervention cohort

The iEDS and clinical judgement labelled 404 IC patients at risk (i.e. 45.6%) (Fig. 2). Two–fifth of these patients (39.1%) were hospitalized on a non-geriatric ward of which more than half (*n* = 89/158; 56.3%) had follow-up by the inpatient geriatric consultation team. One out of four (*n* = 109/404: 27.0%) at risk patients were not hospitalized after the index ED visit. Follow-up by the case manager or the geriatric day hospital was organized for 30 (27.5%) and 13 (11.9%) patients, respectively.

All at risk patients (*n* = 404) had a report of the CGA-based interdisciplinary care plan in the electronic patient record. The geriatric emergency nurse formulated a total of 2772 advices and referrals for at risk patients. On average an at risk patient received seven advices and referrals. Overall, 72.1% of these advices and referrals were adopted completely. The number and adherence of advices and referrals varied from setting to setting: 810 advices and referrals with a complete follow-up of 86.1% concentrated on the index ED stay, 1560 advices and referrals with a complete follow-up of 74.6% focused on the hospitalization following index ED stay and 402 advices and referrals with complete follow-up of 34.1% targeted the post-discharge period. Adherence of post-discharge advices and referrals could not be checked in 23.4% (*n* = 94/402). Table 2 describes the five most reported advices and referrals per setting.

**Table 2:** Top five most reported advices and referrals per setting in at risk intervention patients (*n* = 404)

Most reported advices and referrals during ED admission (*n* = 810 advices in 404 patients)	
1. Advice feasibility of returning home (*n* = 252)	
2. Advice discharge destination (in-hospital or -out-of-hospital) if retuning home was not possible (*n* = 234)	
3. Advice pain management (*n* = 110)	
4. Advice referral to social worker on the ED (*n* = 73)	
5. Advice additional medical follow-up for treating physician on ED (e.g. blood test, technical intervention) (*n* = 69)	
Most reported advices and referrals in case the patient is hospitalized (*n* = 1560 advices in 404 patients)	
1. Advice functional evaluation during hospitalization (*n* = 342)	
2. Advice referral to occupational therapist during hospitalization (*n* = 188)	
3. Advice referral to social worker during hospitalization (*n* = 195)	
4. Advice cognitive evaluation during hospitalization (*n* = 178)	
5. Advice referral to physiotherapist during hospitalization (*n* = 152)	
Most reported post-discharge advices and referrals (*n* = 402 advices in 404 patients)	
1. Advice for additional professional help at home (*n* = 161)	
2. Advice further cognitive evaluation by healthcare workers at home (*n* = 29)	
3. Advice ambulatory follow-up by other medical discipline after ED discharge (*n* = 25)	
4. Advice for (preventive) application for residential care stay (n = 25)	
5. Prescription of aid by physician (e.g. walking aid) (*n* = 24)	

*ED* emergency department

### Unplanned ED readmission (Table 3)

Incidences of unplanned ED readmission in the CC versus IC were 12.1% versus 13.1% at 30 days post-discharge (*p* = 0.28) and 22.1% versus 23.9% at 90 days post-discharge (*p* = 0.11), respectively. Median time to unplanned ED readmission within 90 days post-discharge was 25.1 days in the CC (minimum-maximum: 0.3–88.3 days) and 27.6 days in the IC (minimum-maximum: 0.2–88.0 days) (*p* = 0.66).

**Table 3:** Outcome variables of the URGENT project

Outcome	All patients	Intervention cohort	Control cohort	Unweighted analysis		Weighted analysis^c^	
				Ratio	*P*-value	Ratio	*P*-value
Unplanned ED readmission (at 30 days), N/D (%)	205/1625 (12.6%)	112/857 (13.1%)	93/768 (12.1%)	1.10 (0.85;1.42)	0.49	1.15 (0.89;1.48)	0.28
Unplanned ED readmission (at 90 days), N/D (%)	375/1625 (23.1%)	205/857 (23.9%)	170/768 (22.1%)	1.08 (0.91;1.29)	0.38	1.16 (0.97;1.38)	0.11
Time to unplanned ED readmission within 90 days, days, Me (Min-Max)	26.9 (0.2–88.3)	27.6 (0.2–88.0)	25.1 (0.3–88.3)	0.98 (0.80;1.21)	0.88	0.96 (0.78;1.17)	0.66
Hospitalization rate, N/D (%)	1152/1680 (68.6%)	620/886 (70.0%)	532/794 (67.0%)	1.15 (0.93;1.41)	0.19	1.37 (1.12;1.69)	0.003^a^
Length of ED stay, hours, Me (Min - Max)	16.1 (1.3–110.3)	12.7 (1.4–61.2)	19.1 (1.3–110.3)	0.84 (0.79;0.89)	<.001^a^	0.89 (0.84;0.95)	<.001^a^
Length of inhospital stay, days, Me (Min-Max)	8.7 (0.3–77.5)	8.6 (0.6–76.1)	8.7 (0.3–77.5)	0.97 (0.86;1.09)	0.60	0.92 (0.81;1.03)	0.15
Higher level of care (at hospital discharge), N/D (%)	229/1625 (14.1%)	124/857 (14.5%)	105/768 (13.7%)	1.03 (0.81;1.30)	0.82	1.05 (0.83;1.33)	0.69
Higher level of care^b^ (at 30 days), N/D (%)	37/500 (7.4%)	20/252 (7.9%)	17/248 (6.9%)	1.16 (0.62;2.16)	0.65	1.28 (0.70;2.34)	0.42
Higher level of care^b^ (at 90 days), N/D (%)	36/489 (7.4%)	19/248 (7.7%)	17/241 (7.1%)	1.09 (0.58;2.04)	0.80	1.42 (0.78;2.58)	0.26
Functional decline^b^ (at 30 days), N/D (%)	113/476 (23.7%)	61/236 (25.9%)	52/240 (21.7%)	1.19 (0.86;1.65)	0.29	1.39 (1.01;1.92)	0.04^a^
Functional decline^b^ (at 90 days), N/D (%)	115/479 (24.0%)	63/237 (26.6%)	52/242 (21.5%)	1.30 (0.91;1.86)	0.15	1.51 (1.06; 2.14)	0.02^a^
Post-hospitalization Mortality (at 90 days), N/D (%)	97/1625 (6.0%)	48/857 (5.6%)	49/768 (6.4%)	0.85 (0.56;1.28)	0.44	1.07 (0.71;1.62)	0.73

*ED* emergency department, *Me* median, *Min* minimum, *Max* maximum, *N* Numerator, *D* Denominator

^a^Statistical significant with alpha = .05

^**b**^Data only available for patients without hospitalization after index ED visit

^**c**^Weighted analysis: result of comparison after application of inverse probability of treatment weighting

### Hospitalization and ED LOS (Table 3)

Patients in the IC were more frequently hospitalized compared to CC patients (70.0% versus 67.0%, respectively; *p* = 0.003; significant after Bonferroni-Holm correction). The median ED LOS was 19.1 and 12.7 h in the CC and IC, respectively (*p* < .001; significant after Bonferroni-Holm correction). Median length of inhospital stay was 8.7 days in the CC and 8.6 days in the IC (*p* = 0.15).

### Higher level of care, functional decline and post-hospitalization mortality (Table 3)

Incidence of higher level of care was comparable for both cohorts at all follow-up measurements: approximately 14% at hospital discharge and approximately 7% at both 30 and 90 days. No differences were demonstrated for functional decline between the IC and CC; 25.9% versus 21.7% at 30 days post-discharge (*p* = 0.04; not significant after Bonferroni-Holm correction) and 26.6% versus 21.5% at 90 days post-discharge (*p* = 0.02; not statistically significant result after Bonferroni-Holm correction). Ninety days after hospital discharge, 49 (6.4%) and 48 (5.6%) patients had died in the CC and IC, respectively (*p* = 0.73).

## Discussion

The aim of this study was to evaluate the effectiveness of the URGENT care model that combined CGA-based, interdisciplinary care planning on the ED with geriatric follow-up after ED discharge. Although the current study did not confirm effects of a transitional ED care model on post-discharge outcomes -including the primary outcome; 90-day unplanned ED readmission rate-, IC patients had shorter ED LOS and a higher hospitalization rate.

Previous studies scrutinizing geriatric emergency interventions reported inconsistent effects on unplanned ED readmission rate. Although some were successful, [22–24] other reported no significant reduction in ED readmissions [25–27]. However, comparing existing studies was difficult, since methodological issues (e.g. overall poor study quality and heterogeneity in the targeted populations, intervention strategies and outcomes) limit the evidence base [10]. We hypothesize that the absence of effect on unplanned ED readmission rate (and all other post-discharge outcomes) in the current study is mainly due to low patient acceptance of case management follow-up and low patient adherence to advices in community care. But, off course, many other factors (e.g. social status, cognitive impairment, severity of illness, access to alternative services, missed diagnosis, …) potentially contributed to unplanned readmissions [28]. Although these factors should be attributable to four common themes (i.e. patient, illness, system/organization and clinician), [28] previous studies’ inconsistencies in readmission predictors and limited accuracy of readmission prediction models indicate that the odds of preventing unplanned readmissions in a reliable and efficient way might be small. [29, 30]. Nonetheless, preventing unplanned readmissions should stay a clinical and research objective, because of its importance on macro-, meso- and microlevels. In addition, particularly, early readmissions (i.e. up to 30 days after ED discharge) deserve special attention, as these can be an important determinant of adverse outcomes (e.g. functional decline and mortality) [29].

An important finding of the current study is the reduced ED LOS. Median ED LOS was 6.4 h (− 33.5%) shorter among IC patients, which is clinically relevant for the following two reasons. First, the ED is a hazardous environment for geriatric patients. Shorter ED LOS will prevent ED-stay related adverse events (e.g. pressure ulcer, delirium, falls). Second, it might also improve patient flows through the ED and reduce crowding, which is a high-ranked priority within emergency medicine [31]. Until now, CGA-based interventions in the ED have rarely evaluated its effect on ED LOS [26, 32, 33]. This study shows that despite the CGA by the geriatric emergency nurse followed by interdisciplinary care planning during the ED visit, the total ED LOS was not prolonged as one might assume. On the contrary, it even was reduced substantially.

The increased hospitalization rate among IC patients is another finding that needs further explanation. Especially because this contrasts with previous studies reporting lower hospitalization rates or hospitalization avoidance as main result [23, 26, 27, 34]. The increased hospitalization rate was interpreted as appropriate and necessary, since the assessment for URGENT patients was considered more comprehensive compared to usual care. Indeed, the decision to hospitalize was an interdisciplinary process in which the attending ED physician (and not the geriatric emergency nurse) had the final responsibility. Although comprehensive qualitative research methods were not part of the current study, the study notes that the involved care teams believe that hospitalization avoidance was achieved in several patients as well. The hypothesis that arose from this impression is that the intervention had most likely an impact on the decision-making process of the ED team when carefully balancing the arguments for hospitalization against its alternatives. Unfortunately there were insufficient data available to explore the context and appropriateness of disposition decisions.

Essential to highlight is the need for detailed care process registration in the evaluation of a complex intervention, such as the URGENT care model. Process registration did not only describe how clinical practice was changed. It also explained why intervention effects were present or not. More specifically, the high adherence rate to advices and referrals formulated by the geriatric emergency nurse to be delivered during ED stay (i.e. 86%) explains why outcomes with a direct link to the ED stay (i.e. ED LOS and hospitalization rate) changed significantly, while the opposite occurred in the post-discharge setting (i.e. no impact on post-discharge outcomes due to low patient acceptance of case manager follow-up and low patient adherence to advices in community care (i.e. 34%)). However, despite the process registration in the current study, some results remain difficult to explain. For example, IC patients without hospitalization after index ED visit experienced more functional decline at 30 and 90 days post-discharge. A possible explanation for this result might be the differences in baseline characteristics between the cohorts despite the use of a propensity model.

This study had other limitations which need to be discussed. First, accuracy of the risk stratification component within the intervention was disputable, since the iEDS was a relatively new instrument at the moment of the study, warranting further validation. Its advantage in comparison to classic screening tools was that it allows to target predefined patient strata. Second, selection bias cannot be excluded, since not all patients were included consecutively due to the unpredictable patient flow which is typical for the ED department. In addition, it was difficult to obtain written informed consent among patients with severe cognitive problems. For example, delirium incidence was 3.5 and 4.3% among CC patients and IC patients, respectively, while other ED-based studies reported delirium incidence of approximately 10%. [35, 36] Third, the ED of University Hospitals Leuven moved during the timeframe between both inclusion periods to a new infrastructure. Although the care principles and organization model remained unchanged, this might have influenced the results. Fourth, not every unplanned ED readmission was preventable, while it is obvious that a geriatric care model such as URGENT targets preventable events. However, these could not be documented, because there is no consensus on what is preventable [37–41] Five, it was not possible to conduct subgroup analysis of patients considered at risk for adverse events, since clinical judgement could not be measured in CC patients. Six, future studies can strengthen the evidence base by focusing more on implementation outcomes [42] (e.g. acceptability, fidelity, penetration, costs) and patient/provider reported outcomes (e.g. patient, family and ED staff satisfaction). Seven, as the URGENT care model provided a variety of interventions for two patient groups (i.e. admitted and discharged patients), dilution of intervention effect cannot be excluded. However, subgroup analyses of intervention effect among admitted and discharged patients did not differ from main analyses (data not shown).

The URGENT care model is most transferable to EDs with embedded observation unit or settings that also have the possibility of providing a period of time (i.e. generally up to 24 h or longer) to complete diagnostic tests and initial therapeutic interventions (e.g. assessment units or short-stay units in countries with limited (e.g. 4 h) ED LOS) [43, 44].

## Conclusions

The nurse-led URGENT care model for community-dwelling older patients that comprised CGA-based interdisciplinary care planning on the ED with geriatric follow-up after ED discharge did not reduce unplanned ED readmission rate at 30 or 90 days post-discharge. The most important clinically relevant finding is a substantial decrease of the ED LOS. In addition, the hospitalization rate increased, as well. Although this study implicates that geriatric emergency care models have the potential to improve ED management of older patients, their wide-scale implementation cannot be fully endorsed due to inconsistency of study results. Further research should explore these variations thoroughly.

## Additional file

Additional file 1: Overview of the control cohort. (DOCX 26 kb)

## Data Availability

The dataset of the current study is available from the corresponding author on reasonable request.

## Abbreviations

ADL: Activities of daily living
CAM: Confusion Assessment Method
CC: control cohort
CGA: Comprehensive geriatric assessment
CIRS: Modified cumulative illness rating scale
e.g.: example given
ED: Emergency Department
ESI: Emergency severity index
i.e.: id est.
IADL: Instrumental Activities of Daily Living
IC: intervention cohort
iEDS: interRAI Emergency Department Screener©
ISRCTN: International Standard Randomised Controlled Trials Number
LOS: length of stay
URGENT: Unplanned Readmission prevention by Geriatric Emergency Network for Transitional care

## References

[1] Samaras N, Chevalley T, Samaras D, Gold G (2010). Older patients in the emergency department: a review. Ann Emerg Med.

[2] Kahn JH, Magauran BG, Olshaker SO (2014). Geriatric emergency medicine: principles and practice.

[3] Salvi F, Morichi V, Grilli A, Giorgi R, De Tommaso G, Dessi-Fulgheri P (2007). The elderly in the emergency department: a critical review of problems and solutions. Intern Emerg Med.

[4] Aminzadeh F, Dalziel WB (2002). Older adults in the emergency department: a systematic review of patterns of use, adverse outcomes, and effectiveness of interventions. Ann Emerg Med.

[5] Deschodt M, Devriendt E, Sabbe M, Knockaert D, Deboutte P, Boonen S (2015). Characteristics of older adults admitted to the emergency department (ED) and their risk factors for ED readmission based on comprehensive geriatric assessment: a prospective cohort study. BMC Geriatr.

[6] Carpenter CR, Bromley M, Caterino JM, Chun A, Gerson LW, Greenspan J (2014). Optimal older adult emergency care: introducing multidisciplinary geriatric emergency department guidelines from the American College of Emergency Physicians, American Geriatrics Society, emergency nurses association, and Society for Academic Emergency Medicine. Acad Emerg Med.

[7] Banerjee J, Conroy S, Cooke MW (2013). Quality care for older people with urgent and emergency care needs in UK emergency departments. Emerg Med J.

[8] Rubenstein LZ, Stuck AE, Siu AL, Wieland D (1991). Impacts of geriatric evaluation and management programs on defined outcomes: overview of the evidence. J Am Geriatr Soc.

[9] Ellis G, Gardner M, Tsiachristas A, Langhorne P, Burke O, Harwood RH (2017). Comprehensive geriatric assessment for older adults admitted to hospital. Cochrane Database Syst Rev.

[10] Devriendt E, Conroy S, Nickel C, Bellou A, Conroy S (2018). Comprehensive geriatric assessment in the emergency department. Geriatric emergency medicine.

[11] Devriendt E, Heeren P, Fieuws S, Wellens NIH, Deschodt M, Flamaing J (2018). Unplanned readmission prevention by geriatric emergency network for transitional care (URGENT): protocol of a prospective single Centre quasi-experimental study. BMC Geriatr.

[12] Costa A, Hirdes J, Ariño-Blasco S, Berg K, Boscart V, Carpenter C, et al. interRAI Emergency Department (ED) Assessment System Manual: For Use with the interRAI ED Screener (EDS) and ED Contact Assessment (ED-CA). Washington: interRAI; 2017.

[13] Deschodt M, Flamaing J, Haentjens P, Boonen S, Milisen K (2013). Impact of geriatric consultation teams on clinical outcome in acute hospitals: a systematic review and meta-analysis. BMC Med.

[14] Katz S, Ford AB, Moskowitz RW, Jackson BA, Jaffe MW (1963). Studies of illness in the aged. The index of Adl: a standardized measure of biological and psychosocial function. JAMA..

[15] Tanabe P, Gimbel R, Yarnold PR, Kyriacou DN, Adams JG (2004). Reliability and validity of scores on the emergency severity index version 3. Acad Emerg Med.

[16] Borson S, Scanlan JM, Chen P, Ganguli M (2003). The mini-cog as a screen for dementia: validation in a population-based sample. J Am Geriatr Soc.

[17] Inouye SK, van Dyck CH, Alessi CA, Balkin S, Siegal AP, Horwitz RI (1990). Clarifying confusion: the confusion assessment method. A new method for detection of delirium. Ann Intern Med.

[18] Arroll B, Goodyear-Smith F, Kerse N, Fishman T, Gunn J (2005). Effect of the addition of a "help" question to two screening questions on specificity for diagnosis of depression in general practice: diagnostic validity study. BMJ..

[19] Salvi F, Miller MD, Grilli A, Giorgi R, Towers AL, Morichi V (2008). A manual of guidelines to score the modified cumulative illness rating scale and its validation in acute hospitalized elderly patients. J Am Geriatr Soc.

[20] Rosenbaum PR, Rubin DB (1983). The central role of the propensity score in observational studies for causal effects. Biometrika..

[21] Curtis LH, Hammill BG, Eisenstein EL, Kramer JM, Anstrom KJ (2007). Using inverse probability-weighted estimators in comparative effectiveness analyses with observational databases. Med Care.

[22] Guttman A, Afilalo M, Guttman R, Colacone A, Robitaille C, Lang E (2004). An emergency department-based nurse discharge coordinator for elder patients: does it make a difference?. Acad Emerg Med.

[23] Foo CL, Siu VW, Tan TL, Ding YY, Seow E (2012). Geriatric assessment and intervention in an emergency department observation unit reduced re-attendance and hospitalisation rates. Australas J Ageing.

[24] Caplan GA, Williams AJ, Daly B, Abraham K (2004). A randomized, controlled trial of comprehensive geriatric assessment and multidisciplinary intervention after discharge of elderly from the emergency department--the DEED II study. J Am Geriatr Soc.

[25] Mion LC, Palmer RM, Meldon SW, Bass DM, Singer ME, Payne SM (2003). Case finding and referral model for emergency department elders: a randomized clinical trial. Ann Emerg Med.

[26] Ellis G, Jamieson CA, Alcorn M, Devlin V (2012). An acute Care for Elders (ACE) unit in the emergency department. Eur Geriatr Med.

[27] Keyes DC, Singal B, Kropf CW, Fisk A (2014). Impact of a new senior emergency department on emergency department recidivism, rate of hospital admission, and hospital length of stay. Ann Emerg Med.

[28] Trivedy CR, Cooke MW (2015). Unscheduled return visits (URV) in adults to the emergency department (ED): a rapid evidence assessment policy review. Emerg Med J.

[29] de Gelder J, Lucke JA, de Groot B, Fogteloo AJ, Anten S, Heringhaus C (2018). Predictors and outcomes of revisits in older adults discharged from the emergency department. J Am Geriatr Soc.

[30] Carpenter CR, Shelton E, Fowler S, Suffoletto B, Platts-Mills TF, Rothman RE (2015). Risk factors and screening instruments to predict adverse outcomes for undifferentiated older emergency department patients: a systematic review and meta-analysis. Acad Emerg Med.

[31] Smith J, Keating L, Flowerdew L, O'Brien R, McIntyre S, Morley R (2017). An emergency medicine research priority setting partnership to establish the top 10 research priorities in emergency medicine. Emerg Med J.

[32] Wright PN, Tan G, Iliffe S, Lee D (2014). The impact of a new emergency admission avoidance system for older people on length of stay and same-day discharges. Age Ageing.

[33] Wallis M, Marsden E, Taylor A, Craswell A, Broadbent M, Barnett A (2018). The geriatric emergency department intervention model of care: a pragmatic trial. BMC Geriatr.

[34] Aldeen AZ, Courtney DM, Lindquist LA, Dresden SM, Gravenor SJ (2014). Geriatric emergency department innovations: preliminary data for the geriatric nurse liaison model. J Am Geriatr Soc.

[35] Elie M, Rousseau F, Cole M, Primeau F, McCusker J, Bellavance F (2000). Prevalence and detection of delirium in elderly emergency department patients. CMAJ..

[36] Schnitker L, Martin-Khan M, Beattie E, Gray L (2011). Negative health outcomes and adverse events in older people attending emergency departments: a systematic review. Australas Emerg Nurs J.

[37] McCusker J, Ionescu-Ittu R, Ciampi A, Vadeboncoeur A, Roberge D, Larouche D (2007). Hospital characteristics and emergency department care of older patients are associated with return visits. Acad Emerg Med.

[38] Clarke A (1990). Are readmissions avoidable?. BMJ..

[39] Cardin S, Afilalo M, Lang E, Collet JP, Colacone A, Tselios C (2003). Intervention to decrease emergency department crowding: does it have an effect on return visits and hospital readmissions?. Ann Emerg Med.

[40] Van Walraven C, Jennings A, Taljaard M, Dhalla I, English S, Mulpuru S (2011). Incidence of potentially avoidable urgent readmissions and their relation to all-cause urgent readmissions. CMAJ..

[41] Blunt I, Bardsley M, Grove A, Clarke A (2015). Classifying emergency 30-day readmissions in England using routine hospital data 2004-2010: what is the scope for reduction?. EMJ..

[42] Proctor E, Silmere H, Raghavan R, Hovmand P, Aarons G, Bunger A (2011). Outcomes for implementation research: conceptual distinctions, measurement challenges, and research agenda. Admin Pol Ment Health.

[43] Conroy SP, Ansari K, Williams M, Laithwaite E, Teasdale B, Dawson J (2014). A controlled evaluation of comprehensive geriatric assessment in the emergency department: the ‘Emergency frailty Unit’. Age Ageing.

[44] Ong BS, Van Nguyen H, Ilyas M, Boyatzis I, Ngian VJJ (2012). Medical assessment units and the older patient: a retrospective case-control study. Aust Health Rev.

