# Supplementary material for: Unplanned readmission prevention by a geriatric emergency network for transitional care (URGENT): a prospective before-after study
Source: BMC Geriatr. 2019 Aug 7;19:215. doi: 10.1186/s12877-019-1233-9 (PMC6686568; doi:10.1186/s12877-019-1233-9)
Supplement: Supplementary file 1 — Overview of the control cohort. (DOCX 26 kb) [file 12877_2019_1233_MOESM1_ESM.docx]

**ADDITIONAL FILE 1. Overview of the control cohort**

CONTROL COHORT

n = 794

Hospitalization under supervision of a geriatrician

n = 145 (18.3%)

Hospitalization on non-geriatric ward

n = 387 (48.7%)

Consult by geriatric consultation team during hospitalization (n = 63; 16.3%)

No hospitalization after index ED visit

n = 262 (33.0%)

Visit on geriatric day clinic within 3 months after discharge (n = 17; 6.5%)
